# Unveiling the Role of PEO-Capped TiO_2_ Nanofiller in Stabilizing the Anode Interface in Lithium Metal Batteries

**DOI:** 10.1021/acs.nanolett.2c02973

**Published:** 2022-10-31

**Authors:** Lorenzo Mezzomo, Roberto Lorenzi, Michele Mauri, Roberto Simonutti, Massimiliano D’Arienzo, Tae-Ung Wi, Sangho Ko, Hyun-Wook Lee, Lorenzo Poggini, Andrea Caneschi, Piercarlo Mustarelli, Riccardo Ruffo

**Affiliations:** † Dipartimento di Scienza dei Materiali, Università di Milano Bicocca, 20125 Milano, Italy; ‡ School of Energy and Chemical Engineering, Ulsan National Institute of Science and Technology (UNIST), Ulsan 44919, Republic of Korea; § Consiglio Nazionale delle Ricerche − CNR Istituto di Chimica dei Composti OrganoMetallici − ICCOM, 50019 Sesto Fiorentino (Firenze), Italy; ∥ Department of Industrial Engineering (DIEF) and INSTM Research Unit, University of Florence, Via Santa Marta 3, 50139 Florence, Italy; ⊥ National Reference Center for Electrochemical Energy Storage (GISEL) − Consorzio Interuniversitario Nazionale per la Scienza e Tecnologia dei Materiali (INSTM), 50121 Firenze, Italy

**Keywords:** Solid-state batteries, lithium metal batteries, lithium-ion batteries, ceramic filler, grafted TiO_2_

## Abstract

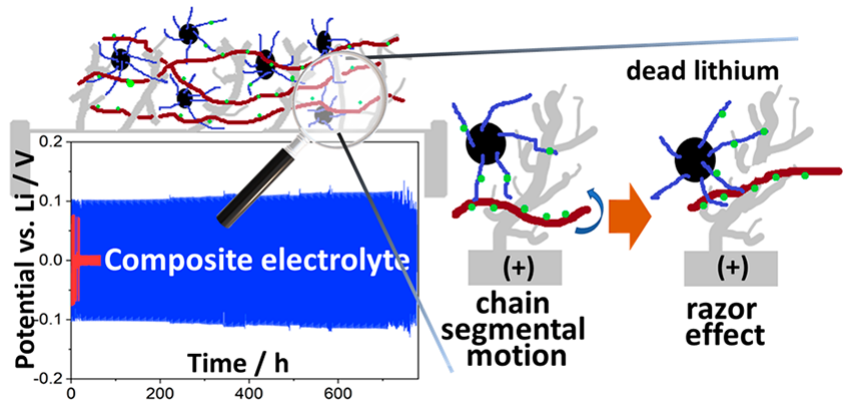

Lithium metal batteries (LMBs) will be a breakthrough in automotive applications, but they require the development of next-generation solid-state electrolytes (SSEs) to stabilize the anode interface. Polymer-in-ceramic PEO/TiO_2_ nanocomposite SSEs show outstanding properties, allowing unprecedented LMBs durability and self-healing capabilities. However, the mechanism underlying the inhibition/delay of dendrite growth is not well understood. In fact, the inorganic phase could act as both a chemical and a mechanical barrier to dendrite propagation. Combining advanced *in situ* and *ex situ* experimental techniques, we demonstrate that oligo(ethylene oxide)-capped TiO_2_, although chemically inert toward lithium metal, imparts SSE with mechanical and dynamical properties particularly favorable for application. The self-healing characteristics are due to the interplay between mechanical robustness and high local polymer mobility which promotes the disruption of the electric continuity of the lithium dendrites (razor effect).

All-solid-state batteries (ASSBs) are among the most promising technologies for successful implementation of safer energy storage devices. In particular, the replacement of flammable liquid electrolytes (LEs) with solid-state ones (SSEs) endows ASSBs with higher safety and lower risk in cases of thermal runaways and short circuits.^1,2^ Additionally, the development of reliable and performant SSEs is playing a major role also in the renewed interest dedicated to lithium metal batteries (LMBs).^3^ Considering the high reactivity of Li with LEs and the severe safety risks due to dendrite growth, the implementation of ASSBs relies on the use of tougher and more electrochemically stable SSEs.^4^ Unfortunately, the use of polymer SSEs such as those based on poly(ethylene oxide) (PEO) does not guarantee an effective action against dendrite propagation due to their intrinsic softness.^5,6^ Consequently, different approaches, such as self-healing capabilities and ceramic blending, have been developed to assess this issue without sacrificing the advantages obtained by the use of polymers such as flexibility and processability.^7−12^

The addition of nanofiller improves the overall toughness of the SSE.^2,13^ However, little attention has been devoted so far to the investigation of mutual interaction between inorganic dispersoids, hitherto considered solely as reinforcing agents, and lithium dendrites. Several ceramic materials used as dispersoids (SiO_2_, TiO_2_) present an elevated reactivity with respect to lithium, which has to be investigated to fully understand the behavior of the whole device.^14−18^ This aspect, almost negligible at low ceramic loadings, is becoming critical due to the possibility of exploiting tailored functionalization processes that enable the incorporation of higher filler contents (>15–20% wt %) into homogeneous, high performance SSE nanocomposites.^19^

Here, the mechanisms underlying the outstanding stability against dendrite penetration of nanocomposite SSEs, described in previous works^20^ and based on the dispersion of PEO_5K_-capped TiO_2_ nanoparticles (PEO_5K_@TiO_2_ NPs) into PEO_4M_ matrix, are thoroughly investigated. The electrochemical performance of the SSE in symmetrical Li/Li cells and in ASSBs is initially recalled, to confirm the literature data and to support subsequent investigations. Then, the focus is placed on titania filler functionalized with short PEG chains, which is fully characterized concerning its reactivity with lithium metal through *in situ* transmission electron microscopy (TEM), electron paramagnetic resonance (EPR), and Raman spectroscopies. Finally, the structural, mechanical, and chemical characteristics of the membrane are correlated with the functional properties by *in situ* Raman spectroscopy, scanning electron microscopy (SEM), X-ray photoelectron spectroscopy (XPS), tensile testing, and time domain nuclear magnetic resonance spectroscopy (TD-NMR). Overall, the reactivity of lithium metal with the capped TiO_2_ nanoparticles is very low, and there is no evidence of titania lithiation. Therefore, the self-healing effects observed in some of the most durable Li|SSE|Li cells must be attributed to the physical interactions among the polymer matrix, the lithium dendrites, and the PEO_5K_@TiO_2_ fillers.

## Electrochemical Performance

**Scheme 1:**
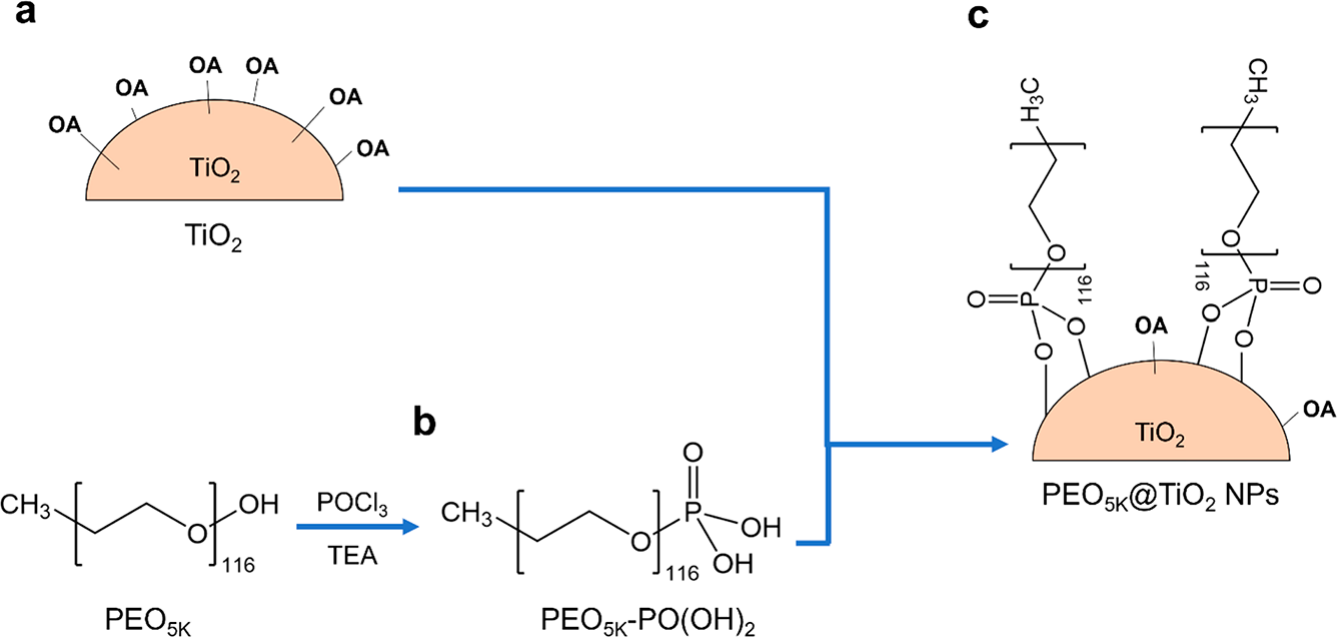
Schematic Representation of PEO_5K_@TiO_2_ Synthesis (a) Production of TiO_2_ NPs capped with oleic acid (OA); (b) functionalization of PEO_5K_ with terminal phosphate group using phosphoryl chloride (POCl_3_) and triethylamine (TEA); (c) direct ligand exchange to achieve the final product.

**Figure 1:**
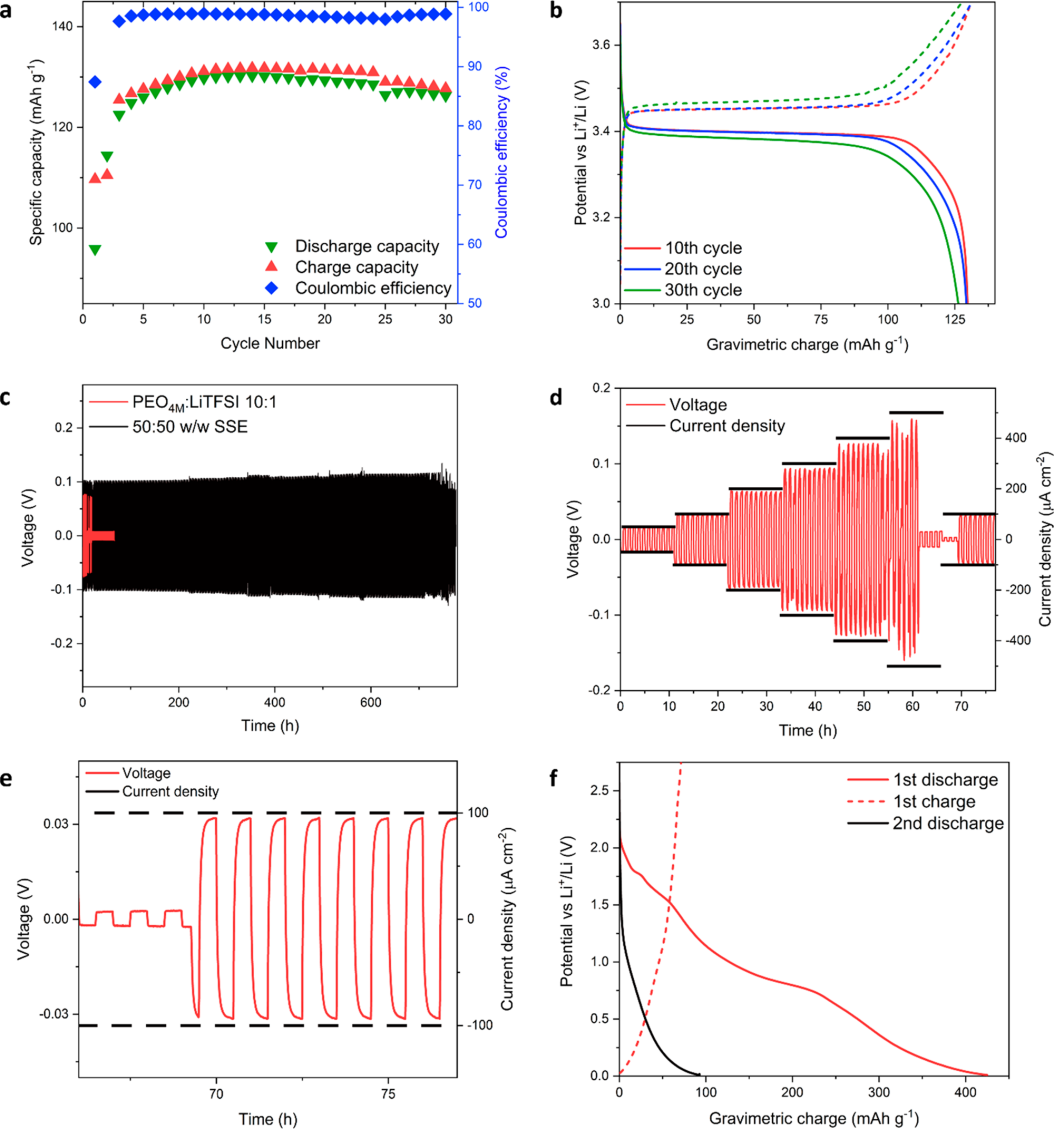
Electrochemical testing of 50:50 w/w SSE and of PEO_5K_@TiO_2_ filler performed at 70 °C. (a) Cycling stability and (b) corresponding voltage profiles of Li|SSE|LFP LMB cycled at C/10. Stripping/plating profiles of Li|SSE|Li (c) at the fixed current density of 200 μA cm^–2^ compared with the result obtained with polymeric analogue and (d) at increasing current density (50–100–200–300–400–500–100 μA cm^–2^) with a magnification (e) on the healed region. (f) Voltage profile of the PEO_5K_@TiO_2_ filler in a half cell vs Li.

As already reported by our group,^20^ PEO_5K_@TiO_2_ fillers can be embedded into a PEO_4M_ polymeric matrix to produce high-performance, long-lasting SSEs for LMBs. The synthesis and main characteristics of these fillers are summarized in Scheme 1 and in Table 1. Additional tests confirmed the ability of the electrolyte made by 50:50 w/w ratio between PEO_4M_ and PEO_5K_@TiO_2_ and containing LiTFSI in the [EO]/[Li] = 10 as conducting salt (50:50 w/w SSE in the following) to operate stably in an ASSB, delivering more than 125 mAh g^–1^ with Coulomb efficiency, CE ≈ 99% (Figure 1a,b). Moreover, 50:50 w/w SSE was capable of sustaining dendrite damage for hundreds of hours (650 ± 150 h obtained on 8 cells) of operation under continuous stripping/plating at 70 °C in Li|SSE|Li cells at a fixed current density of 200 μA cm^–2^ or increasing current densities up to 500 μA cm^–2^ (Figure 1c,d). During these investigations, a peculiar phenomenon was occasionally observed: after the occurrence of dendrite-induced short circuit, noticeable by a sudden voltage drop, most of the cells autonomously reinstated their previous stripping-plating profile (Figure 1e). Similar behavior was not observed when employing purely polymeric electrolytes. Therefore, it is relevant to investigate the mechanisms underlying the performance of the 50:50 w/w SSE, which presumably can disrupt the short circuit initiated by dendrite penetration, eventually leading to the cell restart. Such a mechanism could also be favored by the small particle size and the high operating temperature.^21−23^

**Table 1:** Main Characteristics of PEO_5K_@TiO_2_ NPs

Average size	%TiO_2_	%OA	%PEO_5K_	No. of PEO_5K_ chains	*d*_PEO_	*D*_m_
(nm)^a^	(wt %)^b^	(wt %)^b^	(wt %)^b^	(for NP)	(chain/nm^2^)^c^	(nm)^d^
10.5	53.4	16.6	30.0	154	0.44	1.7

a As determined by Dynamic Light Scattering.

b As obtained by Thermogravimetric Analysis.^20^

c *d*_PEO_: grafting density of PEO_5K_ chains.

d *D*_m_: mean distance between grafted PEO_5K_ chains computed using formula reported by Selli et al.^24^

## PEO_5K_-Capped TiO_2_ Filler Characterization

To evaluate the behavior of the filler under strong reducing conditions, PEO_5K_@TiO_2_-based electrodes were tested in a half-cell against metallic Li between 0.01 and 3.0 V at 70 °C. Although in this case the filler is embedded in a very different chemical environment from that relating to the electrolyte membrane, where the long polymer chains act as electronic insulators, this measurement is intended to demonstrate the possibility of titania lithiation and determine its extent. The results, displayed in Figure 1f, show a specific capacity of 400 mAh g^–1^ after the first reduction. The differential capacity profile (Figure S1) shows the presence of a peak at ∼1.75 V vs Li^+^/Li related to the reversible coexistence of lithium-poor tetragonal Li_*x*_TiO_2_ with orthorhombic Li_0.5_TiO_2_.^25^ However, integration of that peak, normalized for the mass of TiO_2_, indicates a lithiation ratio of about 0.1 Li atoms per TiO_2_ formula unit. Subsequent cathodic processes are attributable to irreversible reductions, formation of SEI, and intercalation of lithium into carbon. The reaction is extremely irreversible, the first anode cycle provides only 95 mAh g^–1^, and subsequent charge and discharge profiles suggest a supercapacitor behavior. Thus, the reactivity of the filler with respect to the lithiation reaction is limited, also because of the presence, on the filler surface, of a non-negligible fraction of oleic acid (OA, > 15 wt %) necessary for an effective displacement reaction with PEO_5K_ and leading to an extremely high particle coverage with the short polymer chains (see Table 1). To obtain TiO_2_ NPs with superior performance for alkaline battery electrodes, capping agents are removed by oxidative processes.^26^

**Figure 2:**
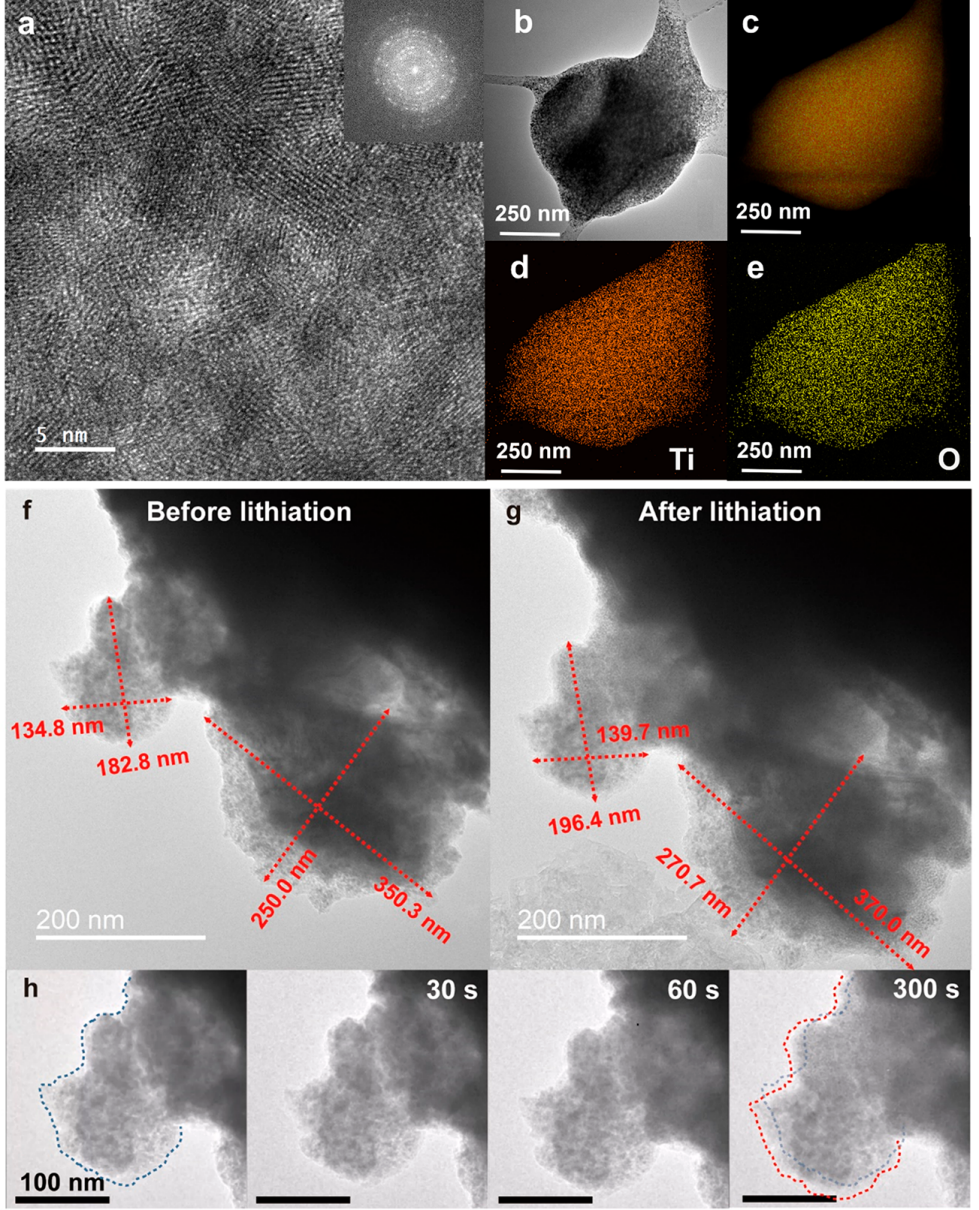
(a) HR-TEM images of PEO_5K_@TiO_2_, with an inset showing the associate selected area electron diffraction pattern. (b–e) SEM (b) and EDS (c) images of PEO_5K_@TiO_2_ highlighting the elemental distribution of (d) titanium (orange) and (e) oxygen (yellow). (f–h) *In situ* TEM images (f) before and (g) after the lithiation of NPs with the (h) corresponding magnified time-lapse evolution of the upper NP. Blue and red dotted lines highlight the edges of the NP before and after the lithiation, respectively.

The reactivity of the filler with lithium was then investigated by electron microscopy (SEM and *in situ* TEM). After having confirmed the polycrystalline nature of PEO_5K_@TiO_2_ (Figure 2a,b) and a homogeneous elemental distribution of Ti and O (Figure 2c–e), further analyses demonstrated that small nanoparticles (NPs) and their resulting agglomerates (<300 nm) present a volumetric expansion of about 11% (Figure 2f–h) when exposed to the lithiation process under an applied relative electrical bias of 1.5 V between Li metal and fillers.

Conversely, as reported in Figure S2, a negligible volumetric increase (∼1%) was noticed for bigger particles (>500 nm). These results confirmed the expected strong influence of the decreasing particle size on the Li storage capacity and possible interaction of Li with NPs;^27^ however, they do not clarify whether the volumetric changes are due to a titania lithiation/conversion process or to the unfolding of polymer chains.

EPR was applied to study the titanium defects in NPs before and after chemical lithiation. The pristine sample (Figure S3a) displays a complex spectrum consisting of two main resonance lines, which were assigned to different O^–•^ and O_2_^–•^ centers, typically detectable in partially oxygen-poor nanometric titania.^28^ The lithiation procedure, besides a partial annihilation of superoxide anions spectral features, leads to an increase of the lower field signal intensity and the appearance of a new resonance at *g* = 1.9910 corresponding to Ti^3+•^ defects (Figure S3a′).^29−31^ Moreover, the subtraction in the low magnetic field range of the resonance features of TiO_2_ (line a) to those of TiO_2_ after lithiation (line a′) unveils the presence of another isotropic signal centered at *g* = 2.0060 ascribable to paramagnetic oxygen vacancies (V_O_^•^ centers, Figure S3b).^29−31^ These results may suggest a partial deprivation of surface lattice oxygen by elemental lithium during the reaction rather than a lithiation process of TiO_2_. Finally, the reaction product between filler and lithium metal was studied with *ex situ* Raman, which showed that there were no substantial differences in the spectra before and after contact with lithium metal (Figure S4). That aspect is discussed at length in the next section, regarding *in situ* measurements on the membrane.

## Solid-State Electrolytic Membrane Characterization

**Figure 3:**
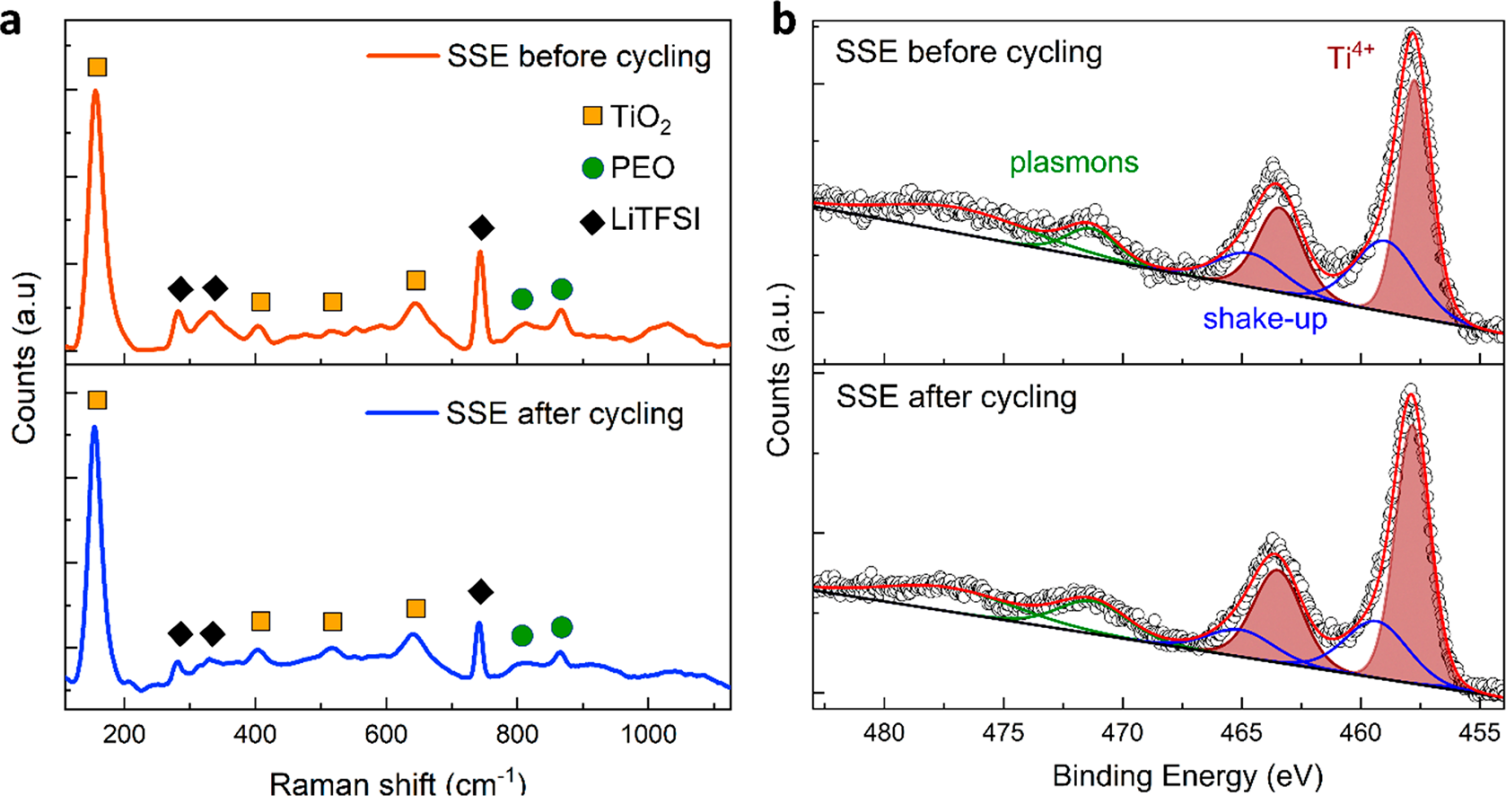
(a) *In situ* Raman spectra of 50:50 w/w SSE before (top) and after (bottom) stripping/plating at fixed current density *j* = 200 μA cm^–2^ in Li|SSE|Li symmetric optical cell. Peaks are ascribed to anatase (orange squares), PEO_4M_ (green circles), and LiTFSI (black diamonds). (b) XPS peaks in the Ti 2p region with the best fit of the SSE before (top) and after (bottom) stripping/plating at fixed current density *j* = 200 μA cm^–2^ in Li|SSE|Li symmetric cell.

*In situ* Raman experiments performed on 50:50 w/w SSE nanocomposite SSE during stripping-plating testing at fixed current densities are reported in Figure 3a. The spectrum of the electrolyte before cycling shows signals assigned to the SSE components, i.e., TiO_2_, PEO, and LiTFSI. The main contribution in the low wavenumber region at 155 cm^–1^ together with secondary features at 404, 520, and 643 cm^–1^ are characteristics of TiO_2_ in the anatase phase and correspond to the E_g_(1), B_1g_(1), B_1g_(2), and E_g_(3) modes, respectively.^32,33^ The Raman analysis also registers the presence of structured bands in the range 150–640 cm^–1^, with secondary peaks at 364 and 584 cm^–1^, which are ascribed to minor contributions from TiO_2_ in the brookite phase.^34−36^ Notably, the main peak at 155 cm^–1^ is shifted approximately 11 cm^–1^ at higher wavenumbers than the expected position for anatase single crystals and shows a larger full width at half-maximum of 24 cm^–1^. This change may be originated from either size-induced phonon confinement or oxygen deficiency.^37−40^ These interpretations are following our TEM and EPR analyses, since the shift value is compatible with nanocrystals ∼5–8 nm in size and/or a TiO_*x*_ composition with *x* ≤ 1.9.^40^ After having performed several stripping-plating cycles on the SSE, all the Raman features relative to TiO_2_ remain unchanged.^41^*In situ* Raman thus confirms that orthorhombic lithium titanate Li_*x*_TiO_2_ is not formed from TiO_2_ during cycling,^42^ thus debunking an important chemical interaction between NPs and lithium.

XPS was applied to evaluate the oxidation state of Ti and to verify the possible NPs lithiation during cell operation. Figure 3b reports the Ti *2p* XPS region for SSE before and after the cycling procedure. The fit of the Ti *2p* XPS spectrum reported in Figure 3b shows the main contribution (wine) at 457.9 eV attributable to Ti^4+^ species.^43−46^ Their relative spin–orbit coupling component (Ti *2p_1/2_*) is shifted by 5.7 eV in line with previous reports.^44−47^ No additional component at lower binding energy indicating the presence of Ti^3+^ was found, disproving again the anatase reaction with Li.^48^ This result does not necessarily contradict what was observed in the EPR spectra taking into account the different sensitivity of the two techniques. EPR has a much higher sensitivity (<1 ppm) than XPS in detecting defect centers (∼1000 ppm). Shake-up components (blue lines) are present at higher binding energies (459.5 and 465.2 eV), and additional satellite features from 471.4 to 477.6 eV (dark green) are observed.^49^ The O *1s* (in FigureS5a) region shows three components: the main peak located at 531.3 eV (filled in blue) corresponds to oxygen bonded with carbon species,^50^ salt,^51^ or Ti–OH bonds^52^ and a lower component at 529.4 eV (wine) corresponding to oxygen atoms bound to Ti^4+^ atoms in the TiO_2_ structure.^44−47^ The Ti/O XPS signal ratio is about 0.6 (considering only the wine component in the O *1s* region) for all samples, confirming that the expected film stoichiometry is retained even after the cycling procedure. The sulfur, as seen in Figure S5b, does not change its chemical behavior and appears as a sulfonyl.^51^ It is worth stressing that after the cycling procedure a small amount of Li^+^ has been found, as reported in FigureS5c, where the peak at 55.1 eV presents a small shoulder given by the Li *1s* due to the presence of partially oxidized dendrites or to the migration of Li^+^ ions.^53^

Overall, most analyses performed on SSEs disproved the hypothesis of dendrite interaction with fillers. Therefore, it was decided to quantify the strengthening effect conferred by filler dispersion by performing tensile tests on three different samples: PEO_4M_ film, PEO_4M_:LiTFSI 10:1, and 50:50 w/w SSE. Apart from the expected amorphization of the polymer observed upon LiTFSI addition coupled with a sharp decrease of the tensile modulus (*E*_T_), Table 2 also highlights the improved mechanical properties achieved by the SSE nanocomposite compared to the fully polymeric analogue.^54,55^ Interestingly, this strengthening effect has not been reported in similar systems for comparable contents (15–20 wt %) of unfunctionalized ceramic fillers due to the poor compatibility between organic and inorganic phases at high loadings.^56^ Moreover, the sharp increase obtained on *E*_T_ was not accompanied by a reduction of the strain at break ε_b_, confirming once again the great homogeneity of the system achieved thanks to filler functionalization.

**Table 2:** Composition, Electrochemical, and Mechanical Properties of Tested Samples

Sample	Organic	LiTFSI	TiO_2_	σ_RT_	σ_70 °C_	*E*_T_	ε_b_	σ_Max_
	(wt %)	(wt %)	(wt %)	(S cm^–1^)^a^	(S cm^–1^)^a^	(MPa)^b^	(%)^c^	(MPa)^d^
PEO_4M_	100	0	0	—	—	200	40	6.1
PEO_4M_:LiTFSI 10:1	60.3	39.7	0	1.0 × 10^–5^	3.4 × 10^–4^	0.9	364	0.1
50:50 w/w SSE	51.4	29.8	18.8	1.2 × 10^–5^	2.9 × 10^–4^	5.3	356	0.5

a Ionic conductivity of solid-state electrolytes respectively at RT and 70 °C.^20^

b *E*_T_: tensile modulus.

c ε_b_: maximum strain at break.

d σ_Max_: stress at maximum load.

**Figure 4:**
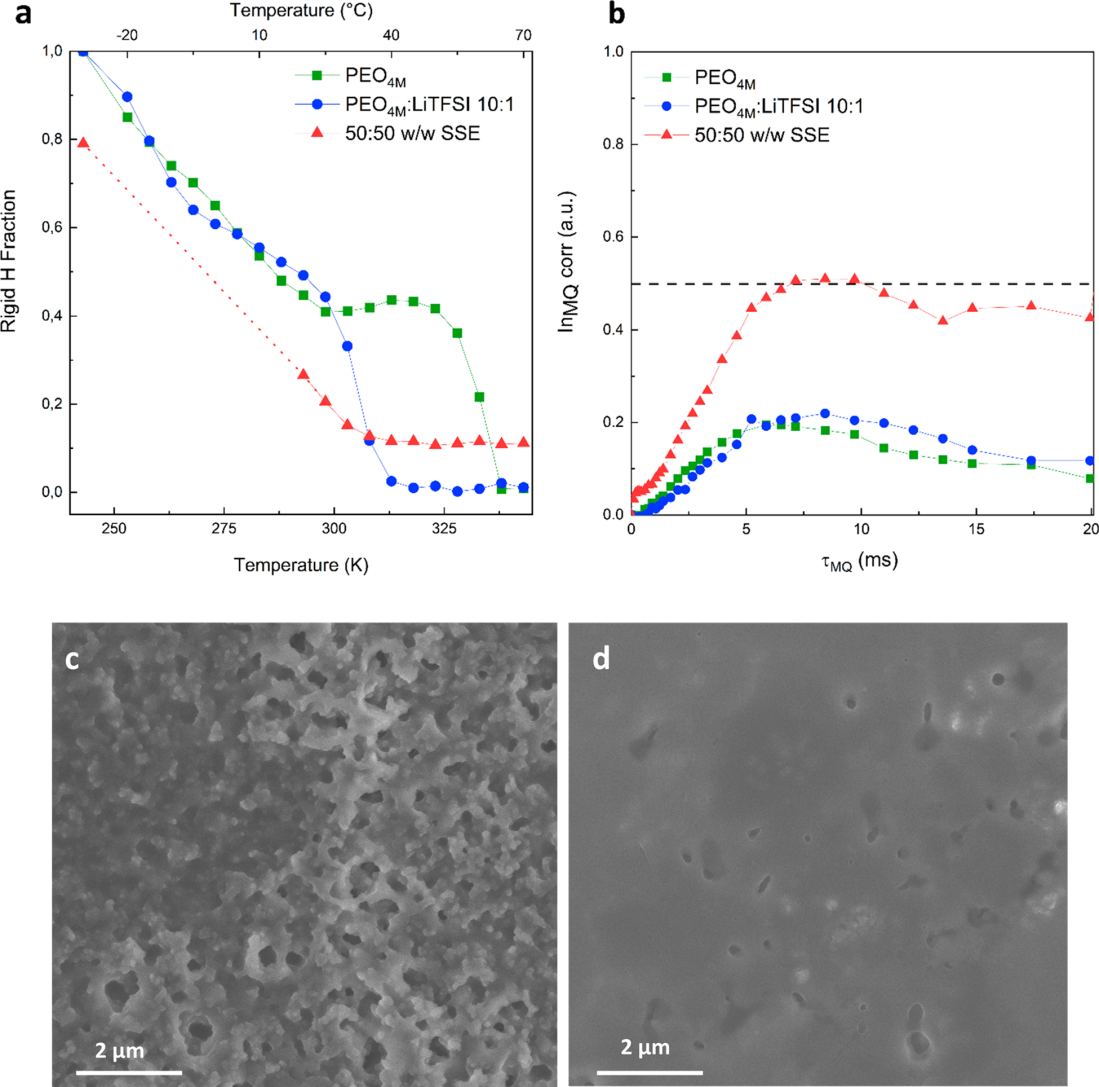
(a) Plot of the rigid fraction of all samples at variable temperature. The dotted section indicates a region where the two component fitting provides unreliable data. Error bars could be added with about 2% uncertainty, which does not alter the detected trends in any way. (b) Normalized MQ NMR build-up curves as a function of the excitation time τ_MQ_ at 343 K, with the 0.5 threshold marked. (c,d) *Ex situ* SEM images of Li anodes after stripping/plating at fixed current density *j* = 200 μA cm^–2^ in Li|Li cells using as electrolyte (c) PEO_4M_:LiTFSI 10:1 and (d) the nanocomposite 50:50 w/w SSE.

Further validation of the aforementioned results was provided by TD-NMR, which can provide fundamental information on chain mobility and morphological features, such as phase distribution and cross-linking.^57^ Here, we recall that PEO_4M_ is a semicrystalline polymer with a crystalline fraction on the order of 70%.^55^ First, by two component fitting of the initial part of the NMR free induction decay after using a magic sandwich echo (MSE) refocusing block, it is possible to quantify the presence of rigid fractions that, being defined by their mobility rather than by structure, in principle can be glassy (if below the glass transition temperature, *T*_g_) or crystalline. As plotted in Figure 4a, PEO_4M_ and PEO_4M_:LiTFSI 10:1 are fully rigid at low temperature and become progressively softer, as seen for samples over *T*_g_,^58^ until they reach *T*_m_ that causes an abrupt decrease of the rigid fraction. As expected, the presence of LiTFSI reduces the melting point by ∼30 K, and the mechanical properties of this sample are indeed consistent with a barely self-standing polymer close to *T*_m_. The evolution of the sample with the further addition of NPs is by far more complicated and the two-component fitting is not effective in the range 250–300 K. Below that temperature, the sample is mostly rigid but retains a small mobile fraction even at 243 K. Interestingly, at *T* > *T*_m_ of PEO_4M_, a non-negligible percentage of rigid polymer (∼10%) is still retained. The behavior in the low–mid temperature range is consistent with the presence of a wide gradient of the motional chain regime chiefly related to the polymer chains connected to NPs due to direct linking or physical absorption, and to the presence of OA.^59^ At *T* > *T*_m_ the OA-PEO_5K_ coverage of NPs favors a chemical interaction with the PEO_4M_ strands, which gives origin to the observed rigid fraction even above *T*_m_.

To obtain information on NPs effect at a less local scale, which is more connected to the mechanical properties, multiple quantum (MQ) NMR with a version of the Baum-Pines (BP) sequence adapted to low field environments was performed at 343 K, i.e., above the melting temperature of the PEO-related crystalline phases, to study the motion of the chains in the absence of crystallites and at the same working temperature of the electrochemical cells. Figure 4b depicts the normalized intensity of the MQ signal, whose build-up correlates with the network behavior of the polymer. It is apparent that samples without NPs do not reach the value of 0.5, predicted for fully developed polymer networks, i.e., systems where significant interchains interactions are active. In contrast, both PEO_4M_ and PEO_4M_:LiTFSI 10:1 display lower values typical of polymer melts. Conversely, the long polymer chains of the 50:50 w/w SSE sample interact with the NPs, likely as physical cross-links, increasing the stiffness of the matrix and explaining the elastomeric behavior of the SSE as a whole. Moreover, for 50:50 w/w SSE it was possible to perform a Tichonov regularization of the build-up curve to extract the underlying distribution *D*_res_ of dipolar couplings, which describes the cross-linking distribution (Figure S6).^60^ The build-up of the MQ signal in Figure 4b is clearly bimodal, with a sharp start and a second slower build-up. Therefore, *D*_res_ shows most of the intensity collected in a sharp peak at low cross-linking as expected for the free-ranging motion of PEO_4M_ and a spike at high (3 kHz) coupling frequencies (Figure S6). This is another confirmation of the presence of strongly constrained polymer chains around the NPs, which act as physical cross-links for the SSE improving the overall mechanical properties.

The effect of the different mechanical properties of the membranes with and without the filler also greatly influences the lithium metal surface during the stripping and plating processes, as can be seen from the SEM images obtained *ex situ* after 5 cycles (Figure 4c,d). The rigid fraction above *T*_m_ results in a more homogeneous distribution of the plating currents and thus a less rough surface in the case of the 50:50 w/w SSE, while in the absence of filler the morphology appears more similar to that obtained in the presence of liquid electrolytes with a significant increase in surface area.^61^

In summary, the physicochemical properties of hybrid ceramic–polymer fillers and of the resulting PEO_4M_-based nanocomposite SSE were systematically investigated, particularly focusing on their behavior with respect to the formation of Li dendrites.

Even if the *in situ* TEM and EPR measurements of PEO_5K_@TiO_2_ demonstrated a small, but non-negligible, degree of interaction with lithium, all the analyses performed on SSE disproved any significant extent of lithiation of dispersed NPs, at least below the sensitivity level of XPS, i.e., from 0.1% to 1%. Conversely, tensile testing and TD-NMR confirmed the beneficial effects imparted by NPs. This effect is not only related to the blending of PEO_4M_ with high-modulus ceramic fillers but also to the cross-linking action endowed by the PEO_5K_ grafted chains. In conclusion, despite the very small reactivity with Li, PEO_5K_@TiO_2_ addition strongly affects the mechanical properties of the SSE and, consequently, the resistance against dendrite propagation.

**Figure 5:**
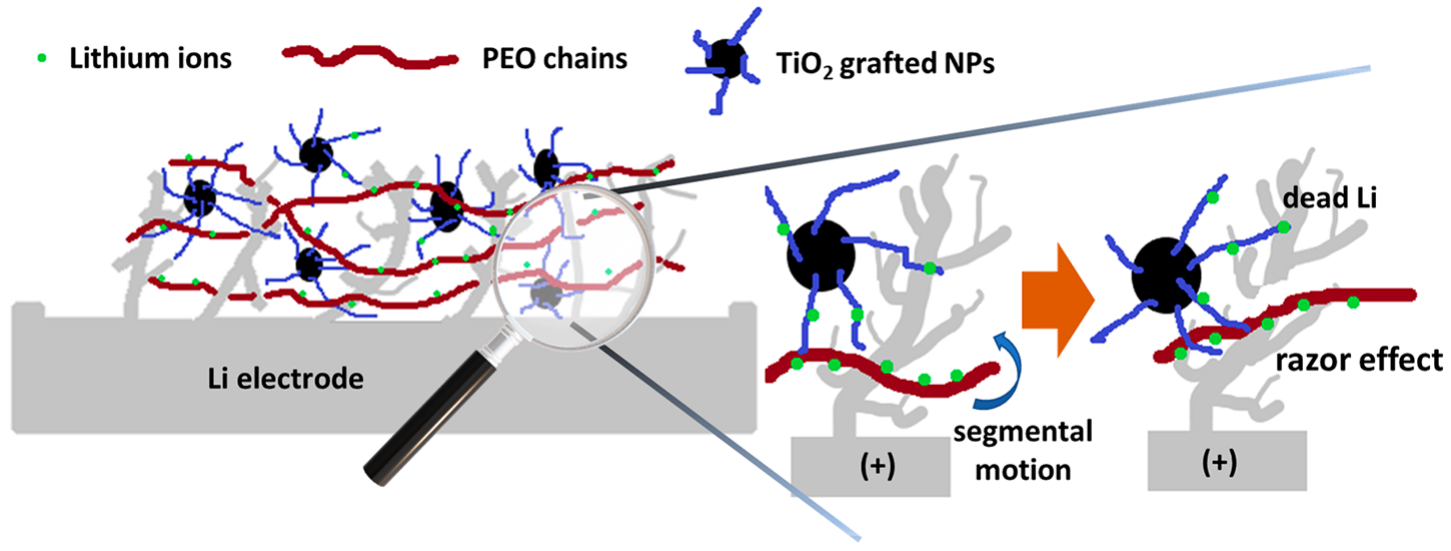
Naive drawing showing the structure of the lithium/SSE interface (left) and detail (right) of the proposed self-healing mechanism, referred to as the “razor effect”.

Finally, we infer that the self-healing properties are due to the interplay between the higher mechanical resistance imparted by the filler and the still high mobility of the polymer matrix, which favors the disruption of the electric continuity of the dendrites and forces lithium to relocate elsewhere in the system as a “dead” metal (razor effect). Figure 5 reports a naive picture of the proposed mechanism, which is nonreversible and causes a progressive increase in the amount of “dead” lithium. In contrast, PEO matrices lacking the necessary amount of filler do not have sufficient mechanical properties to activate this mechanism. Although nonreversible, the intrinsic self-healing mechanism we propose offers solid-state electrolyte designers an important key to the design of systems capable of increasing the lifespan of new-generation ASSBs, without having to resort to complex architectures at the battery management system (BMS) level and/or the use of external stimuli for the activation of chemical/physical repairing agents.

## Supporting Information Available

Experimental details, materials, differential capacity and additional TEM, EPR, Raman, XPS, and NMR results (PDF)
