# Supplementary material for: Unveiling the Role of PEO-Capped TiO2 Nanofiller in Stabilizing the Anode Interface in Lithium Metal Batteries
Source: Nano Lett. 2022 Oct 31;22(21):8509–18. doi: 10.1021/acs.nanolett.2c02973 (PMC9650764; doi:10.1021/acs.nanolett.2c02973)
Supplement: Supplementary file 1 — nl2c02973_si_001.pdf [file nl2c02973_si_001.pdf]

Supporting Information for

UNVEILING THE ROLE OF PEO-CAPPED TIO_2 NANOFILLER IN STABILIZING THE ANODE INTERFACE IN LITHIUM METAL BATTERIES

Lorenzo Mezzomo[a], Roberto Lorenzi[a], Michele Mauri[a], Roberto Simonutti[a], Massimiliano D'Arienzo[a], Tae-Ung Wi[b], Sangho Ko[b], Hyun-Wook Lee[b], Lorenzo Poggini[c], Andrea Caneschi[d], Piercarlo Mustarelli[a,e], Riccardo Ruffo*[a,e]*

a - Dipartimento di Scienza dei Materiali, Università di Milano Bicocca, 20125 Milano, Italy

b - School of Energy and Chemical Engineering, Ulsan National Institute of Science and Technology (UNIST), Ulsan 44919, Republic of Korea

c - Consiglio Nazionale delle Ricerche – CNR Istituto di Chimica dei Composti OrganoMetallici – ICCOM, 50019 Sesto Fiorentino (Firenze) – ITALY

d - Department of Industrial Engineering (DIEF) and INSTM Research Unit, University of Florence, Via Santa Marta 3, Florence, 50139, Italy

e - National Reference Center for Electrochemical Energy Storage (GISEL) - Consorzio Interuniversitario Nazionale per la Scienza e Tecnologia dei Materiali (INSTM), 50121 Firenze, Italy

EXPERIMENTAL SECTION:

Materials: N-Methyl-2-pyrrolidone (NMP, 99.5%), ethylene carbonate (EC, anhydrous 99%), dimethyl carbonate (DMC, anhydrous 99%), lithium bis(trifluoromethanesulfonyl)imide (LiTFSI, anhydrous, 99.99%), 1.0 M $LiPF_6$ solution in EC/DMC (LP30), Lithium (ribbon, 99.9%) and SuperP carbon were purchased from Sigma Aldrich. Polyvinylidene fluoride (PVdF, Solef™ 6020) was acquired from Solvay. PEO_{5K}@TiO_2, PEO_{4M}:LiTFSI 10:1, and 50:50 w/w SSE have been fabricated as reported elsewhere.[20]

Electrochemical measurements: Stripping/plating analyses have been performed both at constant (200 μA cm^{-2}) and variable (50-100-200-300-400-500-100 μA cm^{-2}) current density on symmetric Li|SSE|Li CR2032 coin cells at a fixed temperature of 70°C. For the ex-situ investigation of SSEs, continuous plating analyses at the fixed current density of 200 μA cm^{-2} have been performed to ensure dendrite formation. In these cells, a sandwiched electrolyte configuration with two Celgard® H2010 separators slightly soaked with 50/50 (v/v) solution of EC/DMC 0.5M LiTFSI encapsulating our SSE has been exploited to permit a facile recollection of SSEs, otherwise undetachable from electrodes. PEO_{5K}@TiO_2 NPs electrodes (*ø=16mm*) have been cut from NMP-based slurry composed of 65% wt% of active material, 20% Super P, 15% PVdF coated on copper foil (≈2 mg cm^{-2}) and used as working electrodes into CR2032 coin cells, with Li as counter electrode and LP30 as electrolyte.

These cells have been cycled galvanostatically (200 μA cm^{-2}) between 0.01 and 2.80 V vs Li/Li^+. All the cells have been assembled and disassembled in an MBraun argon-filled glove box (O_2, H_2O < 0.1 ppm) to avoid any contamination. All the electrochemical analyses have been conducted on a BioLogic® VMP3 potentiostat.

Characterization techniques:

High resolution-transmission electron microscopy: (HR-TEM) and energy dispersive x-ray spectroscopy (EDS) analyses of PEO_{5K}@TiO_2 fillers were conducted by using a JEM-2100F TEM (JEOL). An *in-situ* TEM was employed by using a biasing holder (Dual-Probe STM-TEM in situ sample holder, Nanofactory Instruments), which was composed of two electrodes; lithium oxide formed lithium metal on a tungsten (W) tip as a counter/reference electrode and PEO_{5K}@TiO_2 fillers on a copper (Cu) tip as a working electrode to investigate the lithiation process of fillers. An electrical bias of 1.5 V between two electrodes could generate lithium migration from the counter electrode to the working electrode through the thin lithium oxide solid electrolyte generated by about 5 s exposure in the air. The lithiation process was observed until the fillers are fully lithiated inside Tecnai G2 F20 X-Twin TEM (FEI).

Electron Paramagnetic Resonance: EPR investigation on both pristine and lithiated TiO_2 samples was performed by using a Bruker EMX spectrometer operating at the X-band frequency and equipped

with an Oxford cryostat working in the temperature range of 4-298 K. The NPs were charged in quartz glass tubes and spectra were recorded under vacuum conditions ($p<10^{-5}$ mbar) at 130 K. To achieve lithiation, NPs were soaked with an excess of 50/50 (v/v) solution of EC/DMC 0.5M LiTFSI and pressed against two Li foils for 72h into the glove box.

Scanning Electron Microscopy: Lithium anode surface morphology after cycling was evaluated using a Zeiss Gemini 500 scanning electron microscope (SEM) operating at 5kV. To prepare the samples, symmetric Li|Li coin cells cycled at 200 μA cm^{-2} for 10h with the nanocomposite SSE and with the pristine polymeric electrolytes were disassembled in an Ar-atmosphere glove box. Recollected Li anodes were then mounted on SEM stubs using double-sided conductive carbon tape and transferred to the microscope in an argon-filled container. Eventually, they were exposed to air for less than 25s for the loading procedure into SEM.

Micro-Raman: Micro-Raman measurements were performed at room temperature by a confocal LABRAM (JobinYvon) spectrometer operating in a backscattering configuration. *In-situ* measurements were carried out using an ECC-Opto-STD optical cell equipped with an optical glass window and assembled using a symmetric Li|Li vertical configuration and SSE as electrolyte. A small hole (*ø=1mm)* was pierced into the top Li electrode to permit the observation of desired phenomena through the inspection area. A helium−neon laser (λ=632.8 nm, nominal power 17mW) was used as

an excitation source, while scattered light was detected by a Peltier-cooled CCD (Sincerity, JobinYvon). A microscope (Olympus BX40) was used to focus the laser on the sample and to collect the scattered radiation with a long-working distance 50× objective. The spectra are presented after baseline subtraction of broad luminescence signals and the spectrograph position was calibrated with the 521 cm^{-1} reference signal from a Si wafer prior to any measurements.

X-ray Photoelectron Spectroscopy: XPS experiments were carried out in a UHV chamber with a base pressure lower than 10^{-10} mbar. The chamber was equipped with non-monochromatized Al (hν=1486.6 eV) radiation and with a hemispherical electron/ion energy analyser (VSW mounting a 16-channel detector). The operating power of the X-ray source was 150 W (15 kV, 10 mA). Photoelectrons were collected normal to the sample surface and with the analyser maintaining as well the angle between the analyser axis and the X-ray source fixed at 54.5°. All the samples were measured in fixed analyser transmission mode with a pass energy of 44 eV. The binding energy (BE) was calibrated by setting the C *1s* lower component of the Celgard® at 284.0 eV.

Mechanical properties: Tensile experiments were performed at RT on a Zwick Universal Testing Machine using strip-shaped samples of ca. 6.0cm x 1.0cm. Traction was conducted at 500mm/min with preloading of 0.1N using a 100N cell.

Nuclear Magnetic Resonance: TD-NMR experiments were conducted using a Minispec mg20 ND series, with a ^1H resonance frequency close to 19.9 MHz. Temperature control was provided by a BVT3000 unit using a 935 L/h nitrogen flow, cooled by passing through a liquid nitrogen tank for T<300K. MSE and BP sequences are described elsewhere.[56] MSE experiments were performed with 64 or 128 scans, with sufficient recycle delay for full signal recovery, usually 1s. For each sample, the temperature was increased in steps of 5K before repeating the experiments. The longer BP experiments were performed always at 343K, with 128 scans and 42 points.

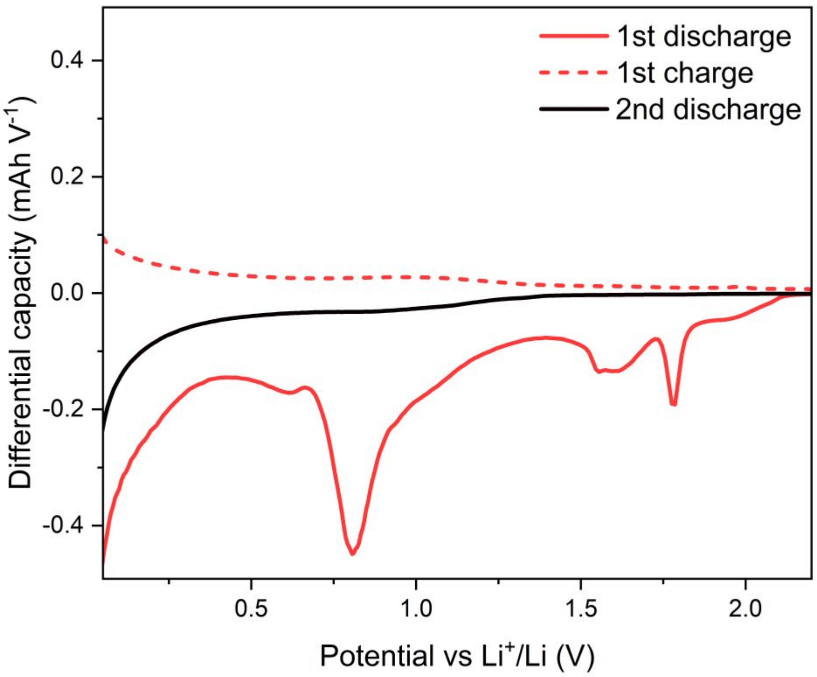

Figure S1. Differential capacity profile of PEO_{5K}@TiO_2 half-cell.

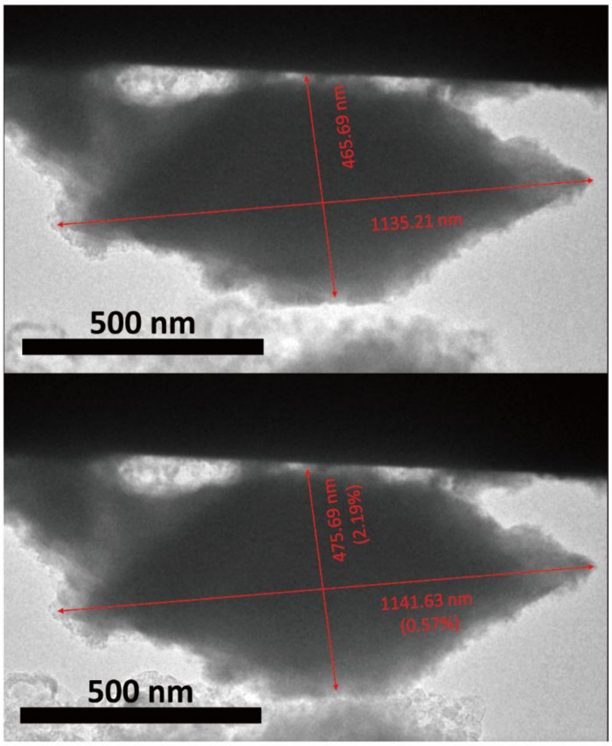

Figure S2. *In-situ* TEM images before and after the lithiation of a big (>500nm) $PEO_{5K}@TiO_2$ particle that shows a reduced volume expansion.

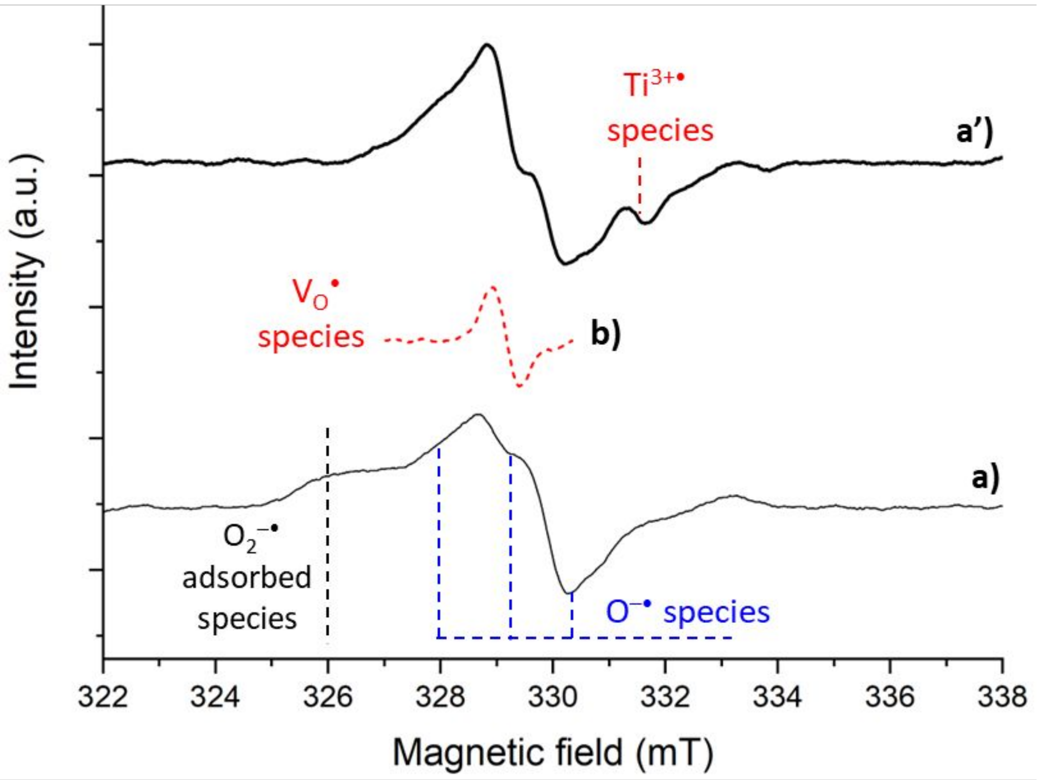

Figure S3. EPR spectra of (a) pristine NPs and (a') lithiated NPs with the (b) resulting subtraction.

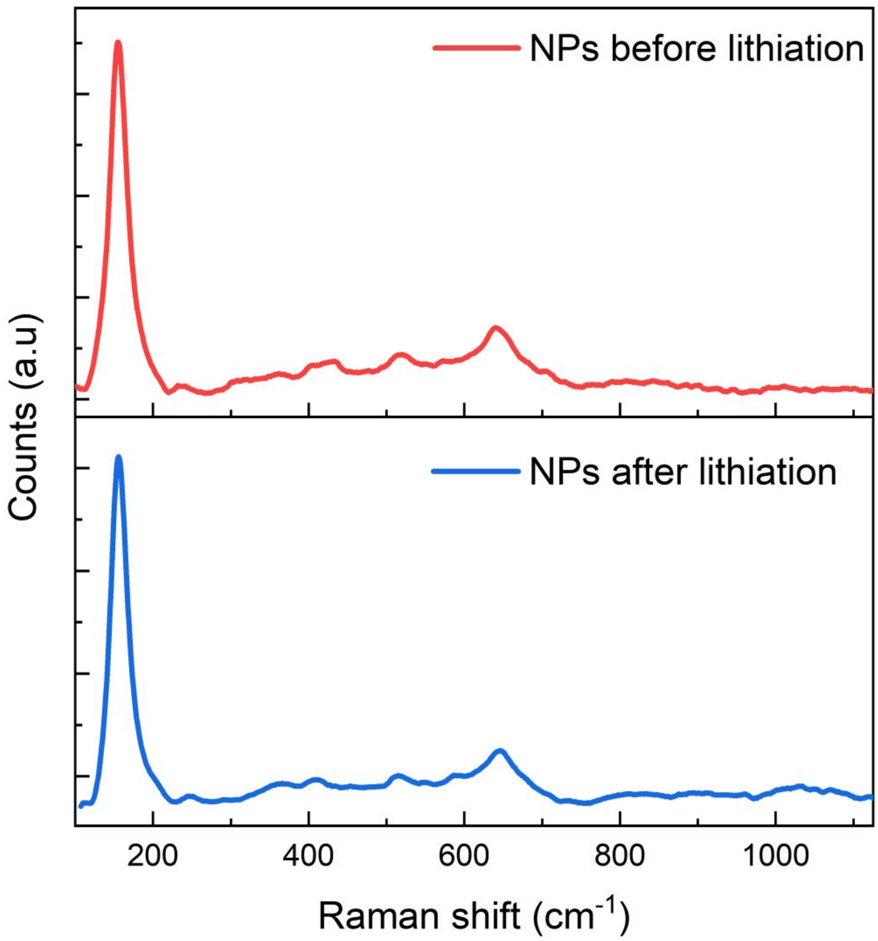

Figure S4. Raman spectra of pristine (top) and chemically lithiated (bottom) TiO_2 NPs.

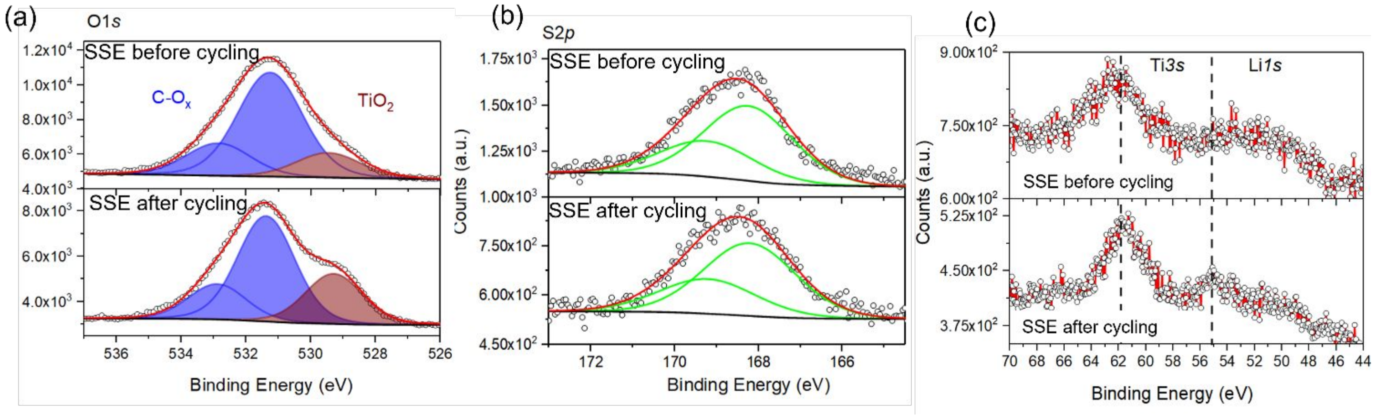

Figure S5. XPS spectra of 50:50 w/w SSE before (top) and after (bottom) the cycling of O *1s* (a) and S *2p* (b) regions and Ti *3s* and Li *1s* (c).

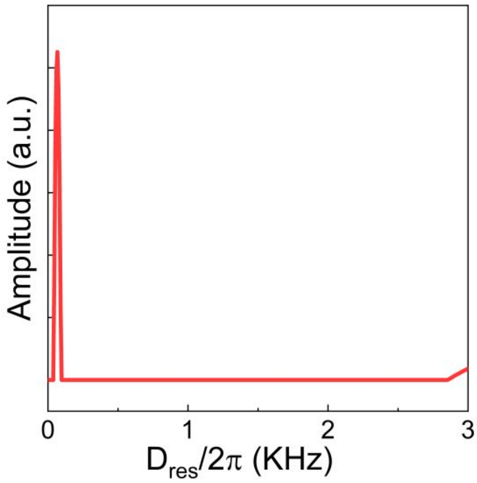

Figure S6. Distribution of dipolar coupling D_{res} obtained by MQ NMR for 50:50 w/w SSE.
